# Service Evaluation of Advice and Guidance Service for Young Adult Mental Health Service in Suffolk

**DOI:** 10.1192/bjo.2026.11526

**Published:** 2026-06-30

**Authors:** Manusmriti Acharya, Abimbola Ojobe

**Affiliations:** Norfolk and Suffolk Foundation Trust, Bury St Edmunds, United Kingdom

## Abstract

**Aims:**

The Advice and Guidance Service for Young Adult Mental Health Service (age 18–25) in Suffolk was established in October 2024 to support GPs with quick and effective treatment advice through a non-face to face platform in managing young adults with mental health difficulties.

No formal evaluation of the service had been conducted since the commencement of the service in October 2024.

The aim of this service evaluation project was to understand the use of the service; its effectiveness and any barriers associated with its use.

**Methods:**

Data around the use of Advice and Guidance service by GP surgeries in both East and West Suffolk region was collected from the eReferral System (eRS) portal.

Specific Evaluation Questions were used to get feedback regarding the awareness and user experience of the A&G service for YAMHS in Suffolk from the GP trainees who work closely with GPs in various GP surgeries in Suffolk.

**Results:**

There were a total of 82 referrals to Suffolk YAMHS via the Advice and Guidance Service and the number of referrals was limited to less than 12 each month.

It was evident that the service was being used by various GP surgeries in both East and West Suffolk.

When looking at the outcomes for these referrals, it was evident that 61 out of total 81 A&G referrals were returned with advice. Among the remaining 20 A&G referrals, 18 were converted into a full referral to the YAMHS team and 2 were returned to the referring GP requesting further information regarding the cases for the team to be able to provide any advice or guidance.

When looking into the barriers for use, unawareness of the existence of the service was a major factor.

An information leaflet was created and sent out to all the GP surgeries in Suffolk via email. Copies of the information leaflet were handed out to the GP trainees attending the West Suffolk GP teaching programme to increase awareness and encouragement towards the service.

**Conclusion:**

The A&G service for YAMHS holds the potential to be an extremely helpful service for the GPs and secondary mental health services in reducing the number of referrals and unnecessary waiting times for cases where quick and effective treatment advice can be appropriately provided through a non-face to face platform.

The barrier in using the Advice and Guidance service for YAMHS was unawareness of the availability of the service among the GPs and their trainees.

We believe that increasing awareness of the service in primary care would greatly increase the use of the service.

